# Seed treatment with macroalgal-derived fucoidan and nanohydroxyapatite mitigates *Fusarium falciforme* ASU26 infection in faba bean: insights from morphological, physiological, anatomical, and FT-IR analyses

**DOI:** 10.1186/s12870-025-06347-7

**Published:** 2025-03-28

**Authors:** Mohamed Gomaa, Eman S. E. Aldaby, Ghada Abd-Elmonsef Mahmoud

**Affiliations:** Botany & Microbiology Department, Faculty of Science, Assiut University, Assiut, 71516 Egypt

**Keywords:** Macroalgae, *Fusarium* control, Nanoparticles, Lignin, Chitinase, Vascular cylinder

## Abstract

**Background:**

Soil-borne diseases are becoming more prevalent due to climate change, while the use of pesticides is being discouraged due to their harmful environmental impacts. This study explored the potential of natural compounds, specifically fucoidan from brown seaweed and nanohydroxyapatite from calcareous red seaweed, as eco-friendly alternatives for mitigating *Fusarium* infections. The treatments aimed to enhance the plant’s defense mechanisms and improve seedling growth.

**Results:**

The treatments using fucoidan, nanohydroxyapatite, or their combination at concentrations of 250–500 µg mL⁻¹ for 6 h, significantly enhanced seedling growth, including increased height, root area, and both fresh and dry weights. Photosynthetic pigment levels and total flavonoid contents increased by more than 30% in treated seedlings compared to the infected control. Malondialdehyde levels, an indicator of oxidative stress, were notably reduced, comparable to or lower than those in the non-infected control. Enzymatic activities associated with plant defense, such as chitinase and polyphenol oxidase, were also higher in treated seedlings. Anatomical improvements were observed, including enhanced vascular cylinder and metaxylem areas. FT-IR analyses highlighted several biochemical changes, such as an increased CH₂/CH₃ ratio indicating lipid structural variation, reduced amide I and II bands, an increase in the C = C band (linked to lignin), and a higher degree of esterification compared to infected controls.

**Conclusions:**

The study demonstrates that fucoidan and nanohydroxyapatite are promising sustainable, cost-effective, and environmentally friendly treatments that effectively boost the defense responses and growth of faba bean seedlings against *Fusarium falciforme* ASU26 infection. These natural compounds could serve as alternatives to conventional pesticides, offering enhanced plant resistance to pathogens and supporting healthier plant growth.

**Clinical trial number:**

Not applicable.

**Supplementary Information:**

The online version contains supplementary material available at 10.1186/s12870-025-06347-7.

## Introduction

Faba bean (*Vicia faba* L.) is a significant legume crop globally, especially in the Middle East and North Africa. Its seeds are valued as a low-cost, nutritious source of food and feed due to their high content of protein (28–30%), carbohydrates (51–68%), as well as minerals and vitamins [1]. Additionally, faba beans are rich in bioactive compounds that offer various health benefits, including hypocholesterolemic, anti-obesity, antioxidant, and anticancer properties [2]. Furthermore, leguminous crops, such as faba beans, contribute to soil health through their ability to fix atmospheric nitrogen via symbiotic bacteria. However, food security is increasingly threatened not only by the growing human population but also by the rising prevalence of soil-borne crop diseases. Additionally, plants are becoming increasingly susceptible to microbial invasion due to continuous exposure to stress and climate change [3].

Several species of the genus *Fusarium* are destructive soil-borne pathogens that affect numerous plants globally, leading to significant crop loss and reduced yields. *Fusarium falciforme* has been reported to cause root rot, with symptoms such as wilting, root browning, decay, and stunted growth in various plant species [4, 5]. To manage and mitigate soil-borne fungal diseases, a range of synthetic fungicides and fumigants are commonly used during plant growth. However, the frequent and indiscriminate application of these chemicals often results in serious negative impacts on both the ecosystem and human health [6]. Consequently, the development of alternative microbial management strategies is crucial for achieving sustainable agricultural practices, both economically and environmentally. The use of natural compounds to directly or indirectly combat plant pathogens is widely encouraged to promote agricultural sustainability. In plants, constitutive and inducible resistance strategies represent two fundamental defense mechanisms against both biotic and abiotic stresses [7]. Constitutive defenses refer to preexisting resistance mechanisms, such as the presence of cuticle, lignin, secondary metabolites, and the formation of structures like hairs or trichomes. Inducible defenses, on the other hand, are activated only after a pathogen attack and may include localized cell death, the accumulation of pathogenesis-related proteins (e.g., chitinases), and cell wall reinforcement through lignin deposition [7].

Algal-derived extracts have been reported to enhance plant growth and either directly or indirectly prevent or alleviate fungal pathogenesis. Their methods of application include foliar spray, soil augmentation, and seed treatment [8, 9]. Seed treatment is a promising technique which triggers a “short memory” effect in plants. This refers to the temporary physiological and biochemical changes in seeds or seedlings, resulting in better responses to biotic and abiotic stresses [8]. Fucoidan, a water-soluble sulphated polysaccharide found in the cell walls of brown seaweeds, has been extensively studied for its bioactivities [10–12]. Seed priming with fucoidan extracts has been shown to increase germination rates and stimulate seedling growth in mung beans [9], finger millet and eggplant [13]. Foliar application of fucoidan has also demonstrated the ability to mitigate salinity stress in wheat seedlings [14]. Moreover, fucoidan oligosaccharides have been reported to trigger immune responses in Arabidopsis seedlings, including oxidative bursts, stomatal closure, and the upregulation of genes related to microbial defense, thereby increasing resistance to *Pseudomonas syringae* pv. *tomato* infection [15].

On the other hand, hydroxyapatite (Ca₁₀(PO₄)₆(OH)₂) is a promising bioactive material, chemically and mechanically similar to human bone and teeth [16]. It is commonly used as a phosphorus source and has shown growth-stimulating effects in some plants [17, 18]. Although hydroxyapatite is typically synthesized chemically, Gomaa and Danial (2023) reported that calcified red algal biomass could serve as a low-cost, sustainable alternative for synthesizing this nanomaterial. Foliar application of nano-hydroxyapatite has been reported to mitigate *Fusarium* wilt disease in tomato [19].

Based on the studies mentioned above, we investigated whether faba bean seed treatment with fucoidan from brown seaweed and phyco-synthesized nanohydroxyapatite could alleviate disease symptoms caused by *Fusarium falciforme*. To our knowledge, no prior study has examined the use of fucoidan and nanohydroxyapatite for seed treatment to protect plants from soil-borne pathogens. Various plant growth and physiological parameters were assessed to evaluate the effects of fucoidan and nanohydroxyapatite on infected plants. Additionally, anatomical and FT-IR analyses of the infected roots were conducted.

## Materials and methods

### Macroalgal biomass

The brown macroalga *Sirophysalis trinodis* (Forsskal) Kützing (formerly known as *Cystoseira trinodis*) and the red macroalga *Liagora viscida* (Forsskål) C. Agardh. were collected from the Red Sea coast, Egypt (Fig. S1). The identification of macroalgae was based on morphological criteria as described by Jha et al. [20] and Algaebase [21]. A household blender was used to pulverize the algal biomass after it had been sun-dried.

### Extraction of fucoidan

The brown macroalga *S. trinodis* was acidified by 2% (w/v) aqueous citric acid at 1.5% w/v under continuous stirring (200 rpm) for 2 h at room temperature. After removing the residual biomass, the extracted fucoidan (FUC) was precipitated using double volume of absolute ethanol. After 24 h at 4 °C, the precipitated fucoidan was collected by centrifugation (3800 g, 10 min) and oven dried. The physico-chemical characterization of fucoidan was carried out as described previously [11].

### Preparation of hydroxyapatite

The calcareous red seaweed (*L. viscida*) was exposed to a 1 M HCl solution for 10 min to produce soluble CaCl_2_ from the insoluble CaCO_3_ in the biomass as follows:

$$\:2\:\text{H}\text{C}\text{l}\hspace{0.17em}+\hspace{0.17em}\text{C}\text{a}\text{C}\text{O}_3\:\to\:\:\text{C}\text{a}\text{C}\text{l}_2\hspace{0.17em}+\hspace{0.17em}\text{C}\text{O}_2\hspace{0.17em}+\hspace{0.17em}\text{H}_2\text{O}$$

The residual biomass was removed by filtration and CaCl_2_ in the filtrate was converted into Ca(OH)_2_ by reacting with NaOH:

$$\:\text{C}\text{a}\text{C}\text{l}_2\hspace{0.17em}+\hspace{0.17em}2\text{N}\text{a}\text{O}\text{H}\:\to\:\:\text{C}\text{a}\left(\text{O}\text{H}\right)_2\:+\:2\text{N}\text{a}\text{C}\text{l}$$

The Ca(OH)_2_ precipitate was collected by centrifugation (3800 g, 10 min), oven dried and utilized for the synthesis of nanohydroxyapatite (nHA).

A standard synthesis of nHA involves mixing of 0.6 M H_3_PO_4_ with Ca(OH)_2_ suspension (1 M) under continuous stirring. The mixture’s pH is regulated at pH 10 using an NH_4_OH solution to prevent the creation of low calcium-apatite [19]. The predicted reaction is as follows.

$$\eqalign{& {\rm{10}}\,{\rm{Ca}}{\left({{\rm{OH}}} \right)_{\rm{2}}}{\rm{ + 6}}\,{{\rm{H}}_{\rm{3}}}{\rm{P}}{{\rm{O}}_{\rm{4}}}\, \to \,{\rm{ + C}}{{\rm{a}}_{{\rm{10}}}}{\left({{\rm{P}}{{\rm{O}}_{\rm{4}}}} \right)_{\rm{6}}} \cr & {\left({{\rm{OH}}} \right)_{\rm{2}}}\,\,\left({{\rm{Hydroxyapatite}}} \right){\rm{ + }}\,{\rm{18}}\,{{\rm{H}}_{\rm{2}}}{\rm{O}} \cr} $$

Centrifugation was used to extract the precipitated hydroxyapatite, which was then oven-dried and ground into a fine powder.

X-ray diffraction (XRD) spectrum of nHA was developed using an X-ray diffractometer (Shimadzu XD-3 A). The morphology of nHA particles was visualized using scanning electron microscope (JEOL JSM 5400 LV) at the Electron Microscopy Unit, Assiut University.

### Fungal pathogen

*Fusarium falciforme* ASU26 was recovered from agriculture soil on potato dextrose agar medium (PDA), the colonies were identified primary based on the morphological characteristics, purified by streaking on PDA medium and maintained at 4℃ on agar slants for short storage and in 40% glycerol at − 20℃ for long storage [22].

The genetical identification of *F. falciforme* ASU26 was performed as described previously [22, 23]. In brief, for DNA extraction, the fungal mycelia from a 5-day-old culture on PDA plates were scratched and transferred to a tube containing 3% CTAB buffer. The mixture was incubated for 30 min at 65 °C, then supplemented with chloroform/isoamyl alcohol and centrifuged for 10 min at 10,000× g. After DNA precipitation with isopropanol, the pellets were washed, supplemented with RNase (10 mg mL^− 1^), and incubated for 30 min at 37 °C. In the final step, the supernatants were discarded, and the pellets were suspended in sterile distilled water for PCR analysis. Universal primers ITS2 and ITS5 were selected for the amplification of the internal transcribed spacer (ITS) region, and the PCR was performed at SolGent, Daejeon Company (South Korea). Sequences of the nearest closely related species belonging to the genus *Fusarium* were downloaded from GenBank, including type specimen sequences. The ITS sequences of *F. falciforme* ASU26 in this analysis were deposited in the National Center for Biotechnology Information (NCBI) with accession number: MK507957. The sequences were analyzed using Clustal W in MegAlign software (version 5.05) for phylogenetic tree construction.

### Growth experiment

Seeds of faba bean (*Vicia faba* L. cultivar Giza 720) were kindly supplied from Agricultural Research Center, Giza 12,619, Egypt. Seeds were surface sterilized for 5 min using sodium hypochlorite solution (4%), followed by washing three times with sterilized distilled water. The seeds were soaked for 6 h in aqueous solutions of fucoidan, nHA, or fucoidan + nHA. Two different concentrations of the compounds were tested viz., 250 and 500 µg mL^− 1^, and the weight ratio of fucoidan and nHA in the mixture treatment were kept at 1:1 (w/w). Seeds soaked in distilled water served as a control. All the seeds were then placed in 9 cm Petri dishes containing 10 mL sterilized distilled water.

For inoculum preparation, *F. falciforme* ASU26 was grown in PDA medium at 28 ± 1 °C for five days, then the conidia were scratched and diluted to 1 × 10^6^ cfu mL^− 1^ in surfactant solution as desperation material (0.1% triton X). The infection was proceeded by adding 100 µL of fungal spore suspension (1 × 10^8^ cfu mL^− 1^) into each Petri dish. The growth experiments (7 seeds per Petri dish) were performed at the greenhouse of Botany and Microbiology Department, Faculty of Science, Assiut University during Winter. Different morphological traits were analyzed after 15 days of germination such as root and shoot length, root collar and stem diameter, and root area were obtained using image analyses using ImageJ 1.54 g software.

### Determination of different physiological parameters of faba bean seedlings

#### Determination of pigments

Leaf samples were extracted using 96% ethanol for 24 h in the dark, and the absorption of the extract was obtained at 470, 649, and 665 nm. The concentrations of chlorophyll a (Chl a), chlorophyll b (Chl b) and carotenoids (CAR) in µg mL^− 1^ were obtained using the following equations [24]:

$$\:Chl\:a=13.95\:{A}_{665}-6.88\:{A}_{649}$$

$$\:Chl\:b=24.96\:{A}_{649}-7.32\:{A}_{665}$$

$$\:CAR=\:\frac{1000\:{A}_{470}-2.05\:Chl\:a-114.8\:Chl\:b}{245}$$

The obtained pigment concentrations were then calculated as mg g ^− 1^ fresh weight (FW).

### Determination of malondialdehyde contents

Lipid peroxidation in the root cells of faba bean was determined as 2-thiobarbituric acid (TBA) reactive substances (malondialdehyde, MDA). Five milliliters of 0.1% (w/v) trichloroacetic acid (TCA) were used to homogenize root samples (0.1 g). The homogenates were then separated by centrifugation (4800 g, 15 min). Root extract (1 mL) was combined with an aliquot of 4 mL reagent (20% w/v TCA and 0.5% TBA aqueous solution), and the mixture was heated for 30 min at 95 ºC. After cooling and centrifugation, the absorbance of the mixture was determined at 450, 532, and 600 nm. The MDA contents were estimated based on the following equation [25]:

$$\eqalign{& MDA \left({\mu mol {g^{ - 1}}FW} \right) \cr & = {{6.45 \times \left({{A_{532}} - {A_{600}}} \right) - \left({0.56 \times {A_{450}}} \right)} \over {Fresh\,weight \left({FW} \right)}} \cr} $$

### Determination of soluble proteins, chitinase and polyphenol oxidase

Fresh roots (0.5 g) were homogenized in potassium phosphate buffer (PB) (50 mM, pH 7) using liquid nitrogen, followed by centrifugation at 4800 g for 10 min. The supernatant was used for the determination of chitinase and polyphenol oxidase (PPO). The protein concentration in the supernatant was measured according to Lowry method [26].

Chitinase (EC 3.2.1.14) was estimated based on the reaction of crude enzymatic extract (200 µL) with colloidal chitin (300 µL) prepared in PB (pH 7.0, 0.1 M), and the reaction was proceeded for 60 min at 37 ºC [27]. The amount of reducing sugars released were estimated using 3,5 dinitrosalicylic acid (DNS) method [28]. A blank for each sample was performed by directly mixing the DNS reagent to the reaction mixture without incubation. Under the assay conditions, one unit of chitinase was defined as the quantity of enzyme that released one µmol of reducing sugars per milliliter in a minute and represented as U mg^− 1^ protein.

PPO was estimated as catechol oxidase (EC 1.10.3.2) using catechol as substrate [29]. The reaction contained crude enzymatic extract (300 µL), PB (1000 µL, pH 7.0, 0.1 M), and catechol (700 µL, 0.05 M). The reaction was incubated at room temperature for 10 min, then terminated using 500 µL of 2.5 N H_2_SO_4_. The blanks were prepared similarly but the reaction was terminated at zero time. One unit of PPO was defined as the quantity of enzyme that causes an increase of 0.1 in absorbance at 495 nm per milliliter in a minute and represented as U mg^− 1^ protein.

### Determination of total phenolics and flavonoids

The extract was prepared by suspending 0.05 of dried roots in 2 mL ethanol for 24 h at room temperature in the dark, followed by adding 1 mL distilled water and extraction at 60 ºC for 30 min, and centrifugation (4800 g, 15 min).

Total phenolic contents (TPC) were measured by diluting 100 µL of the ethanolic extract with 900 µL of distilled water, adding 200 µL of Folin-Ciocalteau reagent, and then adding 400 µL of 20% (w/v) Na_2_CO_3_. After 30 min in the dark, the reaction’s absorbance was measured spectrophotometrically at 750 nm [30]. The amount of phenolics was determined as mg of gallic acid equivalent per gram of dry weight (mg GAE g^− 1^ DW).

The determination of total flavonoids contents (TFC) followed a previous method [30]. The root extract (250 µL) was mixed with 75 µL of NaNO_2_ (5% w/v), and 150 µL of AlCl_3_ (10% w/v). Then, 500 µL of NaOH (1 mM) was added, followed by 2 mL distilled water. The absorbance was measured at 507 nm and the TFC were expressed as mg quercetin equivalent per g dry weight (mg QE g^− 1^ DW).

### Anatomical analysis of plant roots

Transverse Sects. (10–20 μm thickness) of faba bean roots were obtained using a rotary microtome, and then immediately fixed in formalin/acetic acid/alcohol (5/5/90 v/v). The specimens were stained using safranin and light green, then mounted in 1% glycerin, and sealed using DPX. The sections were examined using a light microscope (Olympus CX41 Plan CN, Japan). The cross-sectional area of vascular cylinder and metaxylem vessels were obtained using ImageJ 1.54 g software.

### Fourier transform-infrared (FT-IR) analysis

Fourier transform-infrared (FT-IR) spectra of plant roots were developed using Nicolet ISO 10 FT-IR spectrophotometer. Each FT-IR spectrum was recorded in the wavenumber range of 4000–400 cm^− 1^ at a spectral resolution of 2 cm^− 1^. The spectra were vector normalized, smoothed and baseline corrected. The wavenumber range and position of different functional groups were identified with the help of secondary derivative spectra.

### Statistical analysis

The variations of growth and anatomical parameters were evaluated based on 10 replicates, while other parameters were based on triplicates. All the values were expressed as mean ± standard deviation. The significant differences between treatments at *p* < 0.05 was determined using analysis of variance (ANOVA) followed by post-hoc Fisher’s least square difference test in the GNU PSPP software (version 1.6.2).

## Results and discussion

### Fungal identification

The ITS sequencing of *Fusarium falciforme* ASU26 showed that it has 568 bp with high similarity 99.45% with GenBank accession numbers *F. falciforme* (NR 164424), *F. falciforme* (MG189935), 98.94% with *F. falciforme* (MT251174), *F. falciforme* (MT251175) and (100%) with *F. falciforme* (MH859035), *F. falciforme* (EU329690), while *Clavispora lusitaniae* (JF433045) were deposit as outgroup strain (Fig. S2).

### Characterization of fucoidan

The total sugars, sulphate content, fucose content and molecular weight of extracted fucoidan were 23.20 ± 1.55% w/w, 11.25 ± 1.25% w/w, 9.23 ± 1.23% w/w, and 51.66 ± 2.99 kDa, respectively.

**Fig. 1:**
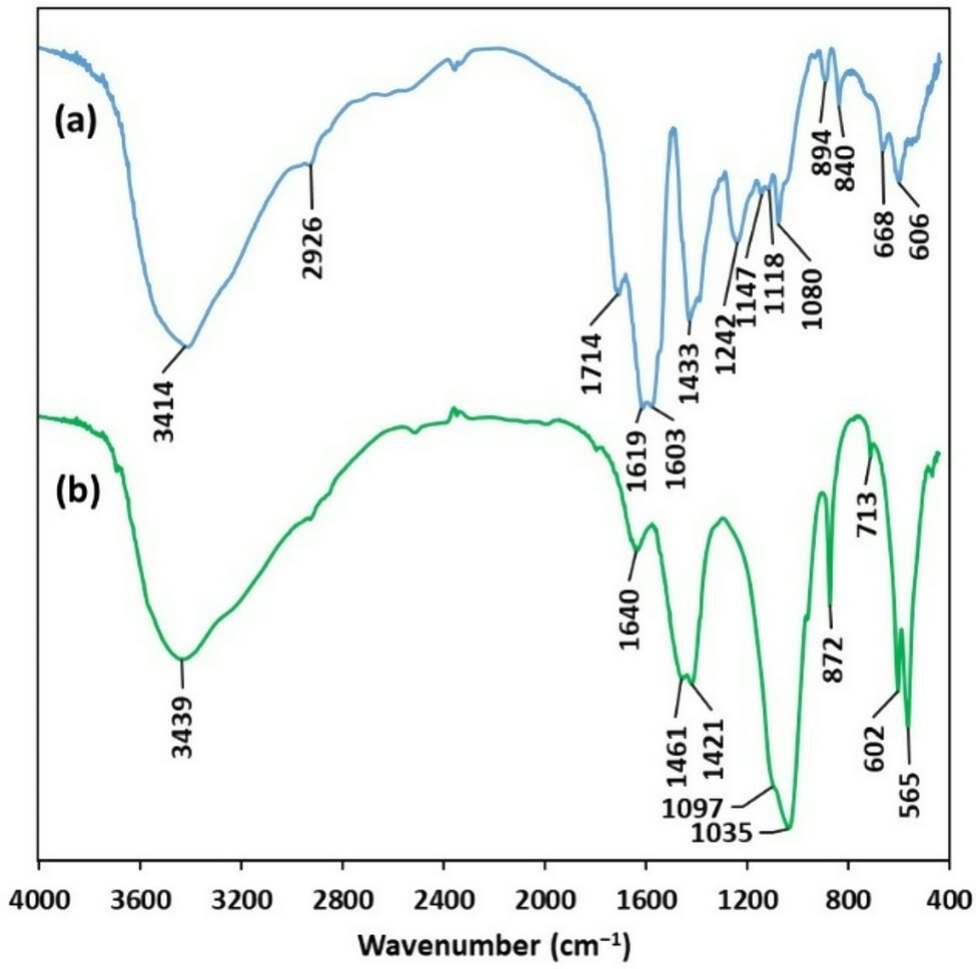
FT-IR spectra of macroalgal derived fucoidan (**a**) and nano-hydroxyapatite (**b**)

The FT-IR spectrum of fucoidan from the brown seaweed *S. trinodis* was depicted in Fig. 1a. The O − H stretching vibration was located at 3414 cm^− 1^. The stretching and bending vibrations of the alkyl groups (–CH_2_−, CH_3_) were observed at 2926 cm^− 1^, indicating the existence of C = O stretching vibrations of O-acetyl groups [31]. The band at 1714 was assigned to the stretching vibration of the C–O of O-acetyl groups [9, 32]. The bands at 1619–1603 cm^− 1^ and 1433 cm^− 1^ were associated with the asymmetric and symmetric stretching vibrations of carboxylate anions (COO⁻) of uronic acids in the fucoidan structure, respectively. The band at 1242 cm^− 1^ indicated sulfate groups (O = S = O stretching). The band at 1147 and 1118 cm^− 1^ were assigned to the stretching of glycosidic linkages (C–O–C) and C–C stretching [33]. The band at 1080 cm^− 1^ was associated with stretching vibrations of C–O and C–OH groups. The sharp band at 894 and 840 cm^− 1^ (C–O–S stretching vibration) indicated the presence of sulfate substitution at C-2 and C-4 position of fucopyranose residues, respectively [31].

### Characterization of nanohydroxyapatite

**Fig. 2:**
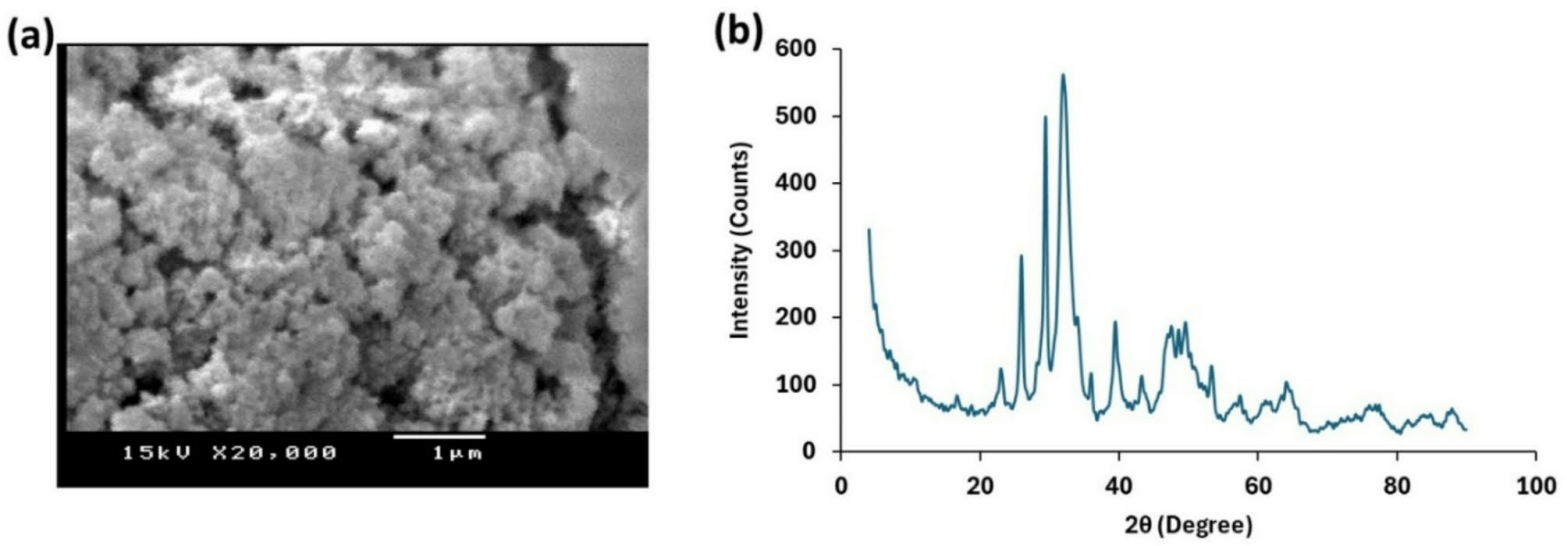
Scanning electron micrograph (**a**) and X-ray diffraction spectrum (**b**) of macroalgal-derived nanohydroxyapatite

Figure 2 depicts the SEM and XRD of macroalgal derived nHA. The SEM indicated an aggregation of nanosized particles. While the XRD analysis confirmed the chemical nature of nHA, where the XRD spectrum was perfectly matched with standard card JCPDS: 01–074–0565. The average crystallite size of nHA was 5.00 nm based on the Debye–Scherrer equation [34]. The FT-IR spectrum of nHA from the calcified red seaweed *L. viscida* indicated the presence of strong and sharp peak centered at 1035 cm^− 1^ (ν_3_ antisymmetric stretching vibrations of the P-O bonds) of PO_4_^3−^ ions (Fig. 1b). The ν₁ symmetric stretching vibration of the phosphate (PO₄³⁻) group was assigned to the shoulder at 1097 cm^− 1^. The stretching vibrations of O − H groups were observed at 3439 cm^− 1^. The bands around 1461 cm⁻¹ and 1421 cm⁻¹ were associated with carbonate (CO₃²⁻) groups (B-type hydroxyapatite) [16]. The splitting sharp peaks at 602 and 565 cm^− 1^ were associated with the ν_4_ bending vibration of the PO_4_^3−^ groups, which occupied two sites in the crystal lattice [35]. The carbonate that occurred in the nHA was attributed to the adsorbed CO_2_ from the air, since the CaCO_3_ in the calcified algal biomass was solubilized using HCl during the first stages of nHA synthesis. The band at 1640 cm⁻¹ was generally associated with the bending vibration of water molecules.

### Effect of treatments on the growth of *Vicia faba*

*F. falciforme* ASU26 infection inhibited the growth of *V. faba* seedlings, with an obvious browning of the rootsFig. . 3). These symptoms are characteristic of *Fusarium* infection, which generally leads to stunted plant growth and root discoloration due to the effects of the pathogen on the root system, resulting in poor nutrient and water uptake [4, 5]. The seedlings height was significantly increased by various treatments, except for the FUC (500 µg mL^− 1^) treatment, compared to the infected control (Fig.  4a). A remarkable increase in shoot length was observed with the FUC (250 µg mL^− 1^) and FUC + nHA (500 µg mL^− 1^) treatments, which were approximately 5-fold longer than the infected control. Furthermore, these treatments were significantly (*p* < 0.001) higher than the non-infected control by more than 1.7-fold. This remarkable enhancement of shoot length not only indicates the potential of these treatments to mitigate the growth-inhibiting effects of the fungus but also suggests a possible stimulatory effect on plant growth beyond the levels observed in non-infected seedlings. Furthermore, the macroalgal-derived compounds helped the infected seedlings restore their original root length as in the non-infected control (Fig.  4a). Similarly, seed priming using water soluble polysaccharides from the brown seaweed *Ecklonia maxima* showed to alleviate *Fusarium oxysporum* f. sp. *lycopersici* infection and enhance plant height and root length [8].

**Fig. 3:**
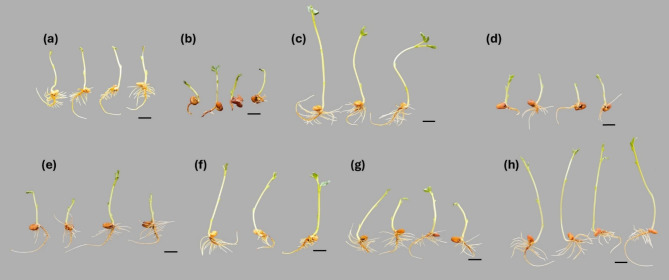
Images of 2-weeks old faba bean seedlings infected with *Fusarium falciforme* ASU26 after different seed treatments. (**a**) non-infected control, (**b**) infected control, (**c**) fucoidan (250 µg mL^− 1^), (**d**) fucoidan (500 µg mL^− 1^), (**e**) nano-hydroxyapatite (250 µg mL^− 1^), (**f**) nano-hydroxyapatite (500 µg mL^− 1^), (**g**) fucoidan + nano-hydroxyapatite (250 µg mL^− 1^), (**h**) fucoidan + nano-hydroxyapatite (500 µg mL^− 1^). Scale bars (3 cm)

**Fig. 4:**
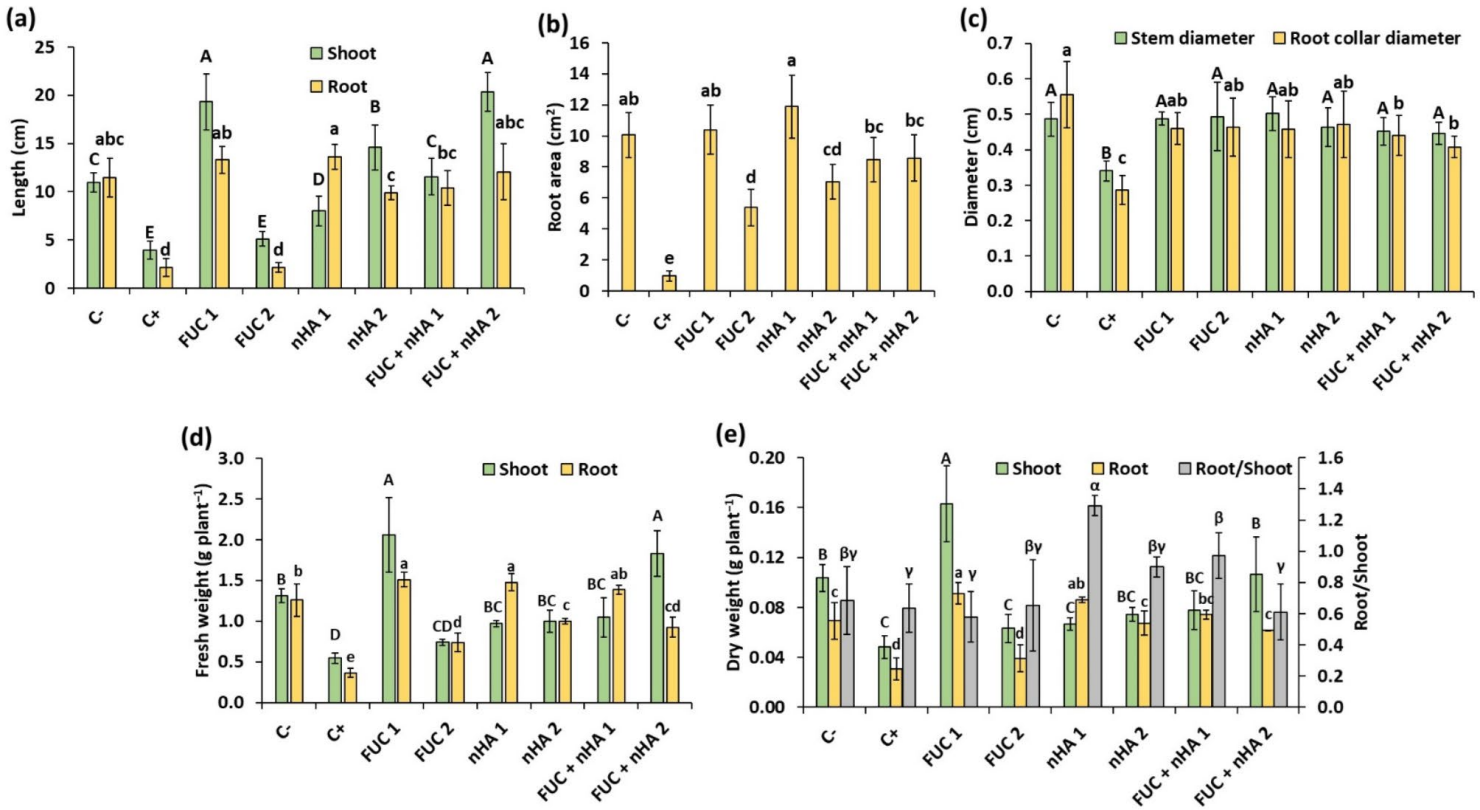
Variations of growth traits of 2-weeks old faba bean seedlings infected with *Fusarium falciforme* ASU26 after different seed treatments. C-: non-infected control, C + infected control, FUC: fucoidan, nHA: nano-hydroxyapatite. The numbers 1 and 2 indicated concentrations of the compounds as 250 and 500 µg mL^− 1^, respectively. Different letters above columns indicate significant differences at p-value < 0.05

The root area was markedly reduced by the fungal infection. Thus, a significant (*p* < 0.001) reduction in root area was observed in the infected control (0.96 ± 0.34 cm^2^) compared to non-infected control (10.05 ± 1.45 cm^2^) (Fig. 4b). FUC and nHA treatments significantly (*p* < 0.01) promoted the root area of diseased seedlings compared to the untreated control. The root area in the treatments was similar to the non-infected control, except FUC (500 µg mL^− 1^) and nHA (500 µg mL^− 1^) treatments.

The fungal infection also reduced stem and root collar diameter, which were reduced by 27 and 48% compared to the non-infected control (Fig. 4c). The application of FUC and nHA significantly (*p* < 0.05) increased stem and root collar diameter compared to the infected control, and the observed values in most of the treatments were similar to the non-infected control.

Overall, the fresh and dry weights of the shoots and roots of faba bean seedlings were markedly reduced by more than 2-fold in response to *F. falciforme* ASU26 infection (Fig. 4d). Seed treatment using FUC and nHA exhibited a remarkable effect on the fresh and dry weights of infected plants in most of the treatments. Seeds treated with FUC (250 µg mL^− 1^) and FUC + nHA (500 µg mL^− 1^) and infected with *F. falciforme* ASU26 showed the highest shoot fresh weights among treatments, estimated to be more than 3- and 1.35-fold higher than the infected and non-infected control, respectively. Similarly, shoot dry weights were significantly higher with FUC (250 µg mL^− 1^) (Fig. 4e). Furthermore, seed treatment with macroalgal-derived FUC and nHA significantly (*p* < 0.001) enhanced the root fresh weights of *F. falciforme* ASU26-infected seedlings by 2- to 4-fold compared to the infected control. Similarly, root dry weights were significantly (*p* < 0.001) increased with different treatments relative to the diseased control (2- to 3-fold increase), except for FUC (500 µg mL^− 1^) treatment (Fig. 4e). The highest increase in root fresh and dry weights was observed with the FUC (250 µg mL^− 1^) and nHA (250 µg mL^− 1^) treatments, which were about 20–30% higher than the non-infected control. The root/shoot dry weight ratios were similar to those of the infected and non-infected controls, except for the nHA (250 µg mL^− 1^) treatment, which showed elevated ratio (Fig. 4e).

These results indicated that macroalgal-derived FUC and nHA seed treatment triggers the plant defense system towards soil-borne pathogens which is consistent with previous studies on different macroalgal extracts and synthetic nHA [8, 19, 36]. However, the treatments exhibited a dose-dependent effect on various growth traits where moderate concentrations (250 µg mL⁻¹) of FUC and nHA provide the most beneficial effects in relation to higher concentrations (500 µg mL⁻¹). Furthermore, the observed effects suggest that while FUC alone at 500 µg mL^− 1^ did not significantly enhance plant growth under *F. falciforme* ASU26 infection, its combination with nHA showed to overcome potential limitations, leading to stress mitigation, and overall plant resilience. This result indicated a degree of synergistic interaction between FUC and nHA.

Since FUC is a highly water-soluble polysaccharide, it can be easily absorbed by seed coatings, allowing for early biochemical responses before germination. Thus, fucoidan acts as a biostimulant by enhancing root growth, plant immunity, and stress tolerance [13]. Furthermore, it contains sulfate groups that may interact with plant signaling molecules, promoting hormone-like effects (e.g., auxin-like activity) [37]. On the other hand, nHA is poorly soluble in water, thus it attaches to the seed coat, forming a thin layer that gradually releases calcium and phosphates. Previous studies confirmed that nHA can be internalized and biotransformed in plant roots into other forms such as α-tricalcium phosphate and β-tricalcium phosphate [17]. Thus, nHA may improve seed hydration and nutrient availability, which is crucial for early root and shoot development. Furthermore, it can modulate stress-responsive genes, helping seedlings resist biotic and abiotic stressors [38].

### Effect of treatments on photosynthetic pigments

**Fig. 5:**
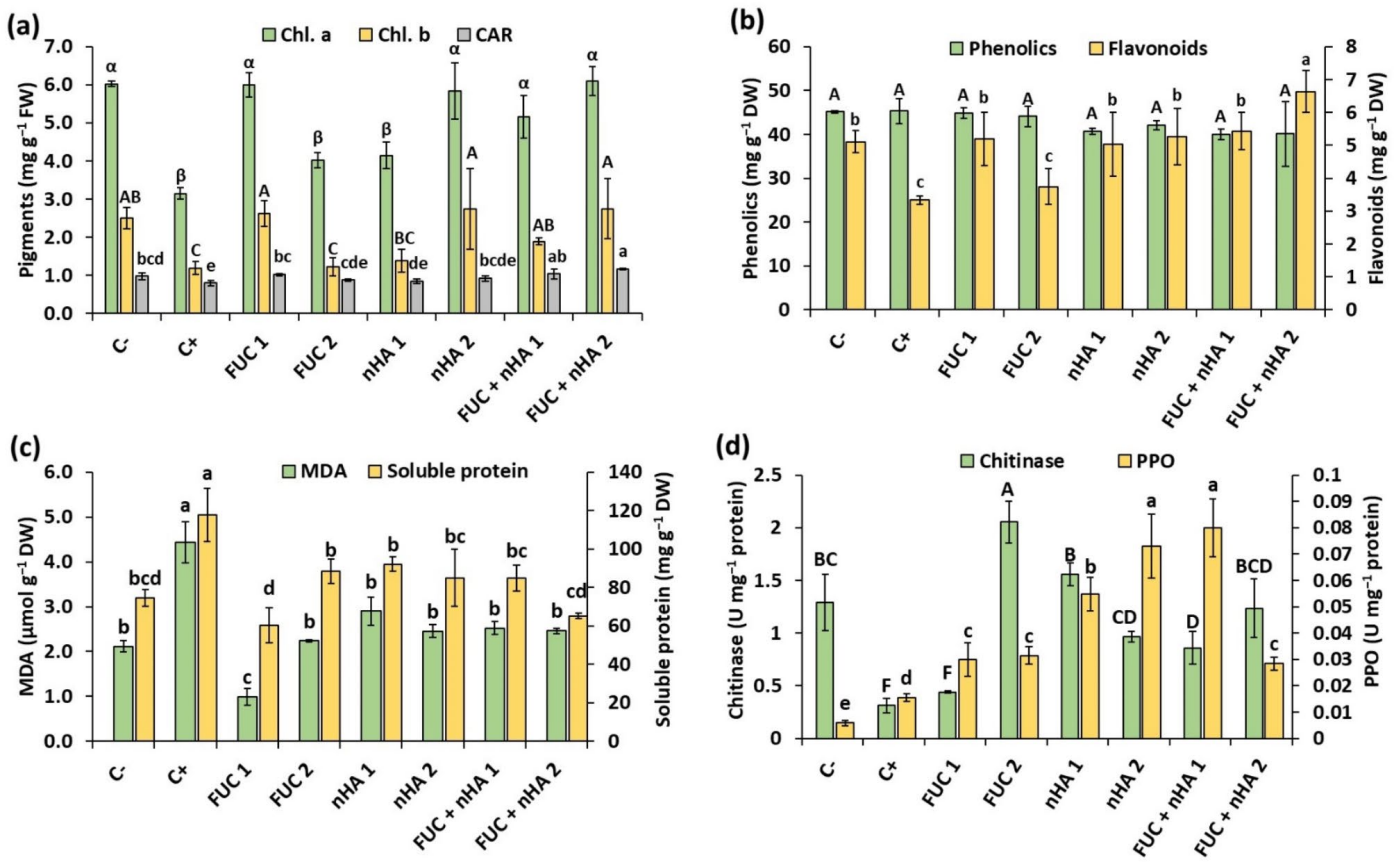
Variations of different physiological traits of 2-weeks old faba bean roots infected with *Fusarium falciforme* ASU26 after different seed treatments. C-: non-infected control, C + infected control, FUC: fucoidan, nHA: nano-hydroxyapatite. The numbers 1 and 2 indicated concentrations of the compounds as 250 and 500 µg mL^− 1^, respectively. chl. a: chlorophyll a, chl. b: chlorophyll b, CAR: carotenoids, MDA: malondialdehyde, PPO: polyphenol oxidase. Different letters above columns indicate significant differences at p-value < 0.05

Infection with *F. falciforme* ASU26 caused more than a 90% reduction in Chl. a and b, and more than a 20% reduction in CAR, compared to non-infected seedlings (Fig. 5a). Seed treatment using FUC (250 µg mL^− 1^), nHA (500 µg mL^− 1^) and their mixtures (250 and 500 µg mL^− 1^) helped the infected seedlings to restore their Chl. a and b contents to those of pathogen-free conditions. Conversely, treatments with mixtures of FUC and nHA significantly (*p* < 0.005) increased CAR contents by 30–45% compared to the infected control (Fig. 5a). These results indicated that *F. falciforme* may have inhibitory effects on pigment biosynthesis in faba been leaves. Furthermore, the accumulation of free radicals during fungal infection caused damage or inhibition in the formation of chloroplasts [39]. Similarly, toxins produced by fungi, especially *Fusarium*, could inhibit pigment biosynthesis in plants. Several toxins from *Fusarium* species such as dihydrofusarubin, fusaric acid, and deoxynivalenol have been reported to induce chlorophyll degradation in plant leaves [40]. Restoring chlorophyll content in treated plants suggests good chloroplast development and improved photosynthetic performance. Similarly, polysaccharides from the brown seaweed *Lessonia nigrescens* enhanced chlorophyll content in wheat seedlings under salt stress [41]. In another study, biogenic nHA was shown to alleviate Cd stress in mung bean seedlings and restore their chlorophyll content [42].

### Effect of treatments on phenolics and flavonoids

The results depicted in Fig. 3b. indicated that the TPC showed no significant differences between treatments. This suggests that faba bean roots maintained relatively constant concentrations of phenolic compounds, which were not directly influenced by the treatments or fungal infection. In contrast, the flavonoid content showed significant variations between treatments. All treatments, except for FUC (500 µg mL⁻¹), significantly (*p* < 0.005) increased (> 30%) the TFC of infected faba bean seedlings compared to the untreated control (Fig. 5b). The highest increase in TFC was observed in the root tissue of the FUC + nHA (500 µg mL⁻¹) treatment. An upregulation of phenylalanine ammonia-lyase, associated with the phenylpropanoid pathway for the synthesis of flavonoids and lignin, was detected in tomato seedlings treated with polysaccharides from macroalgae [36]. The increase in both total phenolics and total flavonoids was detected in *Fusarium* infected plant roots subjected to seed priming using algal-derived polysaccharides [8]. The increase in TFC without a simultaneous increase in TPC in the present study suggests that the phenylpropanoid may have been selectively activated to produce more flavonoids in response to the stress from *Fusarium* infection and seed treatment.

### Effect of treatments on MDA and soluble protein contents

*Fusarium falciforme* ASU26 infection significantly increased MDA and soluble protein content in faba bean roots compared to the non-infected control (Fig.  5c). Seed treatments with FUC or nHA significantly (*p* < 0.001) reduced MDA and soluble protein levels in the infected seedlings, bringing the values similar to or lower than those in non-infected seedlings (Fig.  5c). Elevated MDA levels indicate lipid peroxidation resulting from oxidative stress caused by the accumulation of reactive oxygen species in response to fungal infection. The accumulation of MDA and the associated membrane damage can impair cellular functions, negatively impacting the plant’s growth, water regulation, and nutrient uptake [43, 44].

*Fusarium* infection generally triggers the upregulation of a wide array of stress proteins in plants, including pathogenesis-related proteins, antioxidant enzymes, and other defense-related proteins [45]. The increase in soluble proteins observed in *F. falciforme* ASU26-infected control plants is likely related to the plant’s defense mechanisms (Fig.  5c). These changes are less pronounced in non-infected plants and those treated with FUC and nHA, possibly indicating a lower level of stress. However, other studies have reported a decrease in soluble protein content in infected plants, which may be linked to the inhibition of protein biosynthesis and protein degradation in response to fungal infection [39].

### Effect of treatments on chitinase and polyphenol oxidase activities

Chitinases play a crucial role in plant defense against fungal infections since they catalyzes the breakdown of chitin in the fungal cell walls, thereby inhibiting fungal growth [46]. *F. falciforme* ASU26 infection significantly (*p* < 0.001) reduced chitinase activities to 0.31 ± 0.07 U mg^− 1^ protein, compared to 1.29 ± 0.27 U mg^− 1^ protein in the non-infected control (Fig. 5d). Seed treatment with FUC and nHA promoted chitinase activity in the roots of infected seedlings. The highest increase in the chitinase activity in the roots of faba bean was 2.05 ± 0.20 U mg^− 1^ protein in the FUC (500 µg mL^− 1^) treatment, which was estimated to be 6.6-fold higher than the infected control (Fig. 5d). In contrast, the FUC (250 µg mL^− 1^) treatment showed no significant (*p* > 0.05) variation in chitinase activities compared to the infected control. These observations may indicate that FUC (500 µg mL^− 1^) may upregulate the expression of genes related to chitinase synthesis, however, the lower FUC dose (250 µg mL⁻¹) may not have reached the threshold required for significant induction. Similarly, Righini et al. (2022) observed an increase in chitinase activities in tomato seedlings infected with *Fusarium oxysporum* after seed treatment with higher concentrations of water-soluble polysaccharides from *Jania adhaerens*. In other treatments, using nHA and its mixture with FUC significantly enhanced chitinase activity to 2.8–5.0-fold relative to the infected control (Fig. 5d).

On the other hand, PPO catalyzes the oxidation of phenolic compounds into quinones, which exerts antimicrobial properties. Thus, an increase in PPO activity is directly correlated with disease resistance in plants [47]. Lower PPO activity (0.006 ± 0.001 U mg^− 1^ protein) was observed in the non-infected control compared to other treatments (Fig. 5d). Soaking seeds in the investigated treatments enhanced PPO activity in the *F. falciforme* ASU26-infected plant roots (0.028–0.080 U mg^− 1^ protein) compared to the infected control (0.016 U mg^− 1^ protein) (Fig. 5d). The highest increase in PPO was observed in the FUC + nHA (250 µg mL^− 1^) and nHA (500 µg mL^− 1^) treatments, which were approximately 5-fold higher than the infected control. An enhancement in PPO was also detected in wheat seedlings drenched in liquid brown algal extract after infection with *Fusarium graminearum* [48].

### Anatomical features of *Vicia faba* root

**Fig. 6:**
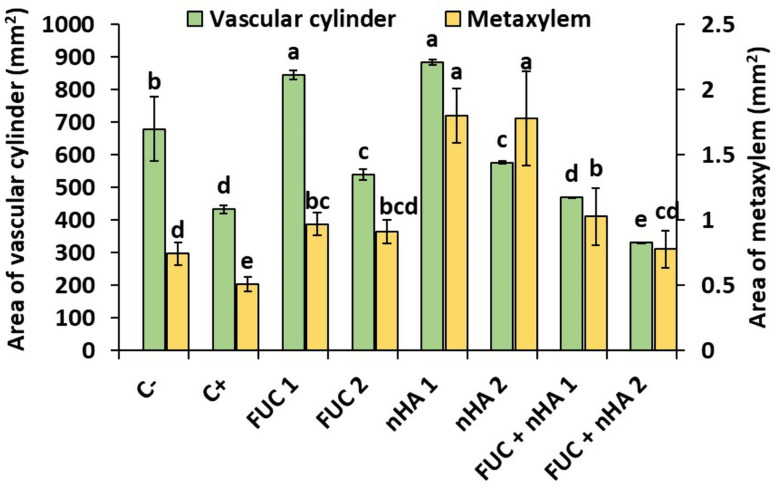
Variations of cross-sectional area of vascular cylinder and metaxylem vessels in 2-weeks old faba bean roots infected with *Fusarium falciforme* ASU26 after different seed treatments. C-: non-infected control, C + infected control, FUC: fucoidan, nHA: nano-hydroxyapatite. The numbers 1 and 2 indicated concentrations of the compounds as 250 and 500 µg mL^− 1^, respectively. Different letters above columns indicate significant differences at p-value < 0.05

The cross-sectional area of the vascular cylinder in a plant root plays a crucial role in water and nutrient transport. Similarly, larger metaxylem vessels have higher hydraulic conductance, ensuring efficient water and minerals transport from the root to the stem. *Fusarium* spp. infection generally results in a narrower and less efficient vascular cylinder, impairing the root’s ability to transport essential resources [49]. The present results confirmed the adverse effects of *Fusarium* infection on the cross-sectional area of both vascular cylinder and metaxylem vessels, which were reduced by > 30% in relation to the non-infected control (Fig. S3, Fig. 6). The application of nHA and fucoidan enhanced the root development, which was reflected in promotion of the cross-sectional area of both vascular cylinder and metaxylem vessels (Fig. 6). The single treatment of the plant seeds with 250 µg mL^− 1^ of nHA or FUC promoted the cross-sectional area of vascular cylinder by > 94% in relation to the infected control and by > 24% compared to the non-infected control. The other treatments, except nHA + FUC (250 µg mL^− 1^) treatment, exhibited significant enhancement of the cross-sectional area of vascular cylinder in relation to the infected control (Fig. 6). On the other hand, the nHA treatment (250–500 µg mL^− 1^) resulted in the highest improvement of the cross-sectional area of metaxylem, which was estimated to be ~ 2.5-fold higher than the infected control and ~ 1.4-fold higher than the non-infected control. Similarly, other single or mixed treatments were able to enhance the cross-sectional area of metaxylem vessels compared to the infected control and their values were significantly higher or similar to the non-infected control (Fig. 6).

### FT-IR analysis of plant roots

The normalized FT-IR spectra of faba bean roots were depicted in Fig. S4. The spectra were analyzed for main changes in the lipid, protein, and carbohydrate regions.

### Changes in the lipid region

**Fig. 7:**
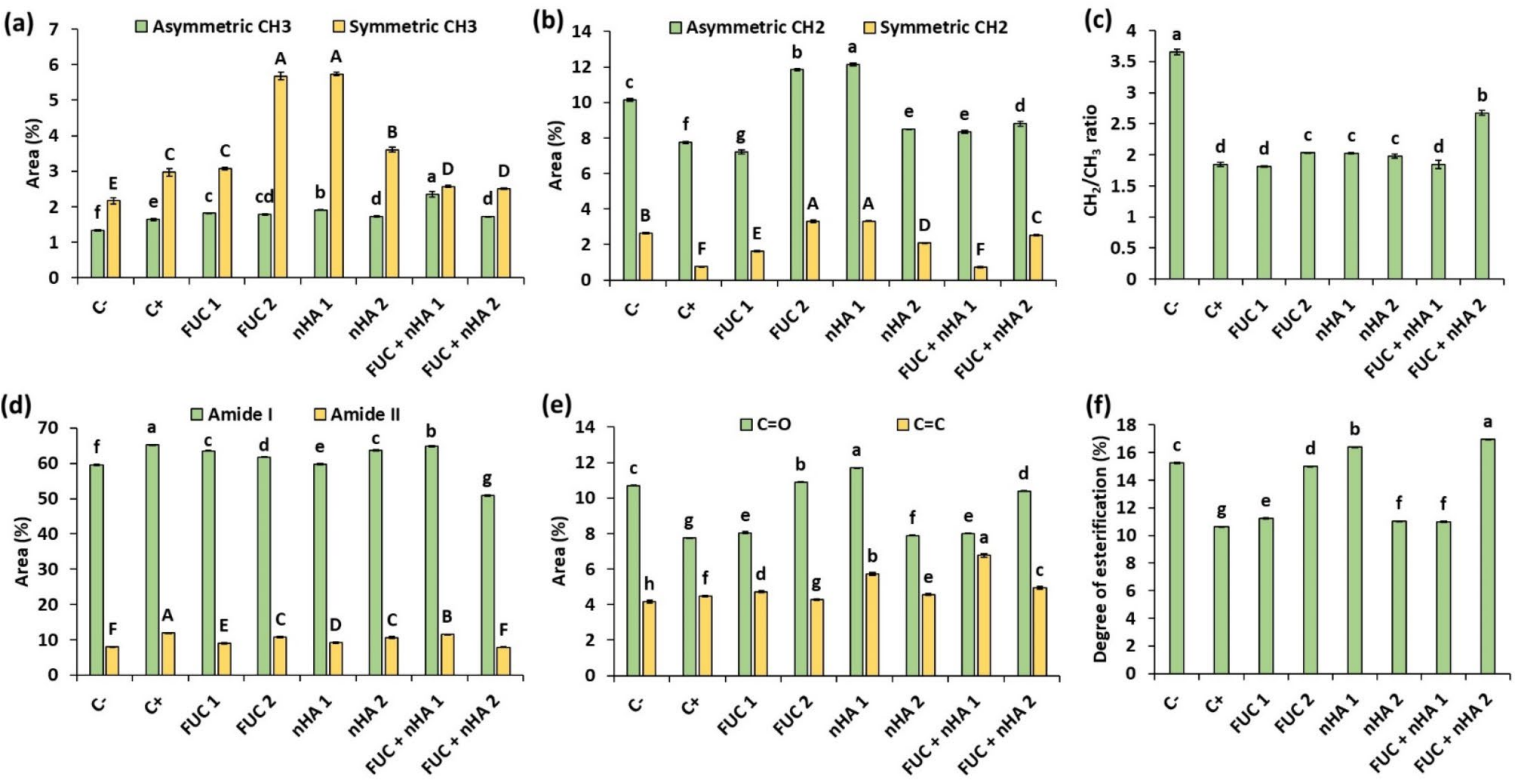
Peak area analyses of different spectral regions in the FT-IR of 2-weeks old faba bean roots infected with *Fusarium falciforme* ASU26 after different seed treatments. C-: non-infected control, C + infected control, FUC: fucoidan, nHA: nano-hydroxyapatite. The numbers 1 and 2 indicated concentrations of the compounds as 250 and 500 µg mL^− 1^, respectively. Asymmetric CH_3_: 2990–2945 cm^− 1^, asymmetric CH_2_: 2945–2905 cm^− 1^, symmetric CH_3_: 2885–2860 cm^− 1^, symmetric CH_2_: 2860–2840 cm^− 1^, amide I: 1700–1600 cm^− 1^, amide II: 1560–1525 cm^− 1^, C = O: 1760–1720 cm^− 1^, C = C: 1525–1505 cm^− 1^, degree of esterification = C = O area/(C = O area + amide I area). Different letters above columns indicate significant differences at p-value < 0.05

The FT-IR bands located at 3000–2800 cm^− 1^ are related to the CH_3_ and CH_2_ groups of lipids and certain amino acids side chains. The asymmetric and symmetric stretching vibrations of CH_3_ groups were detected in the spectral region 2990–2945 and 2885–2860 cm^− 1^, respectively [50]. The area of bands related to asymmetric CH_3_ groups in all treatments were significantly (*p* < 0.05) higher than infected and non-infected control, and FUC + nHA (250 µg mL^− 1^) treatment exhibited the highest increase (Fig. 7a). Conversely, the symmetric CH_3_ groups were significantly (*p* < 0.05) increased in the treatments: FUC (500 µg mL^− 1^) and nHA (250 and 500 µg mL^− 1^) compared to other treatments (Fig. 7a). The CH_3_ groups in faba bean roots were generally lower under normal growth conditions. On the other hand, the peaks at 2945–2905 and 2860–2840 cm^− 1^ were assigned to the asymmetric and symmetric stretching vibrations of CH_2_ groups [50]. The area of CH_2_-related bands was significantly (*p* < 0.05) reduced in the infected plant roots compared to most of the treatments, and higher values were observed in FUC (500 µg mL^− 1^) and nHA (250 µg mL^− 1^) treatments (Fig. 7b). Generally, the increase of CH_3_ and CH_2_ groups in treatments suggests that seed treatment could upregulate lipid metabolism, induce membrane restructuring, or cause an increase in the presence of methylene-rich compounds. Furthermore, the increase in symmetric CH_2_ indicated higher membrane fluidity [51]. This possibly induced more resilient or responsive plant seedlings to fungal infection and enhanced plant growth. The increase of biosynthesis of phospholipids, glycolipids, and sphingolipids in the plasma membrane and lipid-derived metabolites is mainly related to the plant immune signaling during microbial infection [52]. Additionally, changes in the symmetric CH_2_/CH_3_ ratio reflect the length of the aliphatic chain and degree of branching and saturation of lipid-related metabolites [53]. Fungal infection significantly (*p* < 0.001) reduced the CH_2_/CH_3_ area ratio compared to non-infected control, but most of seed treatments produced roots with significantly (*p* < 0.001) higher CH_2_/CH_3_ area ratio compared to non-infected plants (Fig. 7c). The decrease in CH_2_/CH_3_ area ratio may indicate that the fungal infection altered lipid biosynthesis in infected plant roots, leading to more branched or shorter lipid molecules. Furthermore, fungal infection accelerates lipid oxidation and breakdown of polyunsaturated fatty acids into short chain saturated fatty acids, leading to lowering their CH_2_/CH_3_ ratio [54]. This result implied more oxidative damage and membrane disruption in the infected control, which agreed with the results of MDA contents (Fig. 5c).

### Changes in the protein region

The spectral region at 1560–1525 cm^− 1^ indicated amide II band from a combination of N-H bending and C-N stretching vibrations of proteins. While the peaks in the 1700–1600 cm^− 1^ region are specific to the amide I band, which are mainly associated with the C = O stretching vibration of proteins with little contribution of in-plane N–H bending and C-N stretching vibrations [50, 53]. The peak area analysis indicated that fungal infection significantly (*p* < 0.05) increased the area under amide I and II bands compared to the non-infected control and different treatments (Fig. 7d). These observations implied markedly higher protein contents in the infected control compared to other treatments, which support the results of soluble protein content. An increase in the amide I band was also detected in rachis of susceptible wheat cultivar after *Fusarium graminearum* infection [55]. In contrast, Righini and coworkers observed that *Fusarium oxysporum* reduces the amide I and II area in the FT-IR spectrum of tomato roots, while seed priming using *Ecklonia maxima-*derived polysaccharides increased the relative area of these bands [8]. Seed treatment using FUC and nHA in the present study significantly reduced the amide I and II bands in infected plants compared to the non-primed infected control. These variations are generally related to different defense responses and adaptation mechanisms between different plants.

### Changes in the carbohydrate region

The band located at 1760–1720 cm^− 1^ was assigned to the stretching vibration of carbonyl ester groups (C = O), which are commonly found in pectin and lipids [56]. The degree of esterification in plant roots can be calculated as the percentage peak area ratio between the C = O groups and the sum of C = O groups and amide I band [52, 56]. The FT-IR analyses indicated significant (*p* < 0.001) reductions in the C = O groups and degree of esterification in control seedlings infected with *F. falciforme* ASU26 compared to other treatments (Fig. 7e, f). The decrease in the C = O groups implied lower esterified compounds in infected roots. Pectin is generally de-esterified through the activity of fungal pectinases such as pectin methylestrase, which facilitate the establishment of infection [46]. Similarly, Lahlali and coworkers observed a reduction in the C = O groups in wheat rachis infected with *F. graminearum* [55]. The degree of esterification also determines the sensitivity of plants to microbial infections, where high content of pectin methyl esterification is associated with promoted plant resistance [57]. Accordingly, the increased degree of esterification in faba bean after seed treatment with FUC and nHA indicates cell wall strengthening and less susceptibility to degradation by pathogens.

The peaks in the spectral region 1525–1505 cm^− 1^ indicates C = C stretching vibrations of the aromatic ring of lignin [58]. The integrated area analysis of this region indicated significantly (*p* < 0.001) higher values in the infected control compared to the non-infected control (Fig. 7e). This result implied higher lignin contents which is related to the upregulation of lignin biosynthesis, as one of the main responses to fungal attack [59]. The application of FUC and nHA seed treatment further promoted the C = C area in all treatments, except FUC (500 µg mL^− 1^) (Fig. 7e). Seed priming using FUC and nHA promoted lignin accumulation in the infected plant roots, thus most of the treatments had higher peak area at the 1525–1505 cm^− 1^ compared to the non-treated controls. Similarly, soaking of date palm roots in fucoidan solutions from brown macroalgae showed to stimulate the natural defenses by eliciting the accumulation of lignin [60]. In another study, the application of fucoidan to the leaf discs of olive tree was reported to increase the levels of phenolic compounds and lignin. In contrast, Righini and coworkers indicated that seed priming using fucoidan did not increase the lignin contents of tomato seedlings infected with *F. oxysporum* in relation to the infected control [8].

## Conclusion

This study confirmed that seed treatment with macroalgal-derived fucoidan and nano-hydroxyapatite enhanced plant defense responses against *Fusarium falciforme* ASU26 infection. Low concentrations (250 µg mL^− 1^) of fucoidan and nano-hydroxyapatite, as well as their combination at 500 µg mL^− 1^, improved various growth traits in infected faba bean seedlings. Seed treatments resulted in a physiological status closely resembling that of non-infected plants, as indicated by higher levels of photosynthetic pigments, flavonoids, chitinase, and polyphenol oxidase, along with reduced malondialdehyde content compared to untreated controls. Notably, individual treatments with fucoidan and nano-hydroxyapatite were more effective than their combined treatment in promoting the cross-sectional area of the vascular cylinder and metaxylem vessels, suggesting enhanced water and nutrient transport from root to stem. Furthermore, FT-IR analysis showed an increased CH_2_/CH_3_ ratio in lipids for most treatments, along with a notable decrease in the amide I and II bands compared to infected controls. The analysis also confirmed an increase in the C = C band associated with lignin and a higher degree of esterification in infected roots following seed treatment. Field validation of these effects is crucial. The use of macroalgal-derived fucoidan and nano-hydroxyapatite offers a sustainable and eco-friendly strategy for managing fungal diseases, potentially reducing the hazardous impacts of synthetic fungicides.

## Electronic supplementary material

Below is the link to the electronic supplementary material.

Supplementary Material 1

## Data Availability

The datasets used and/or analyzed during the current study are available from the corresponding author on reasonable request.
