# Supplementary material for: Seed treatment with macroalgal-derived fucoidan and nanohydroxyapatite mitigates Fusarium falciforme ASU26 infection in faba bean: insights from morphological, physiological, anatomical, and FT-IR analyses
Source: BMC Plant Biol. 2025 Mar 28;25:394. doi: 10.1186/s12870-025-06347-7 (PMC11951647; doi:10.1186/s12870-025-06347-7)
Supplement: Supplementary file 1 — Supplementary Material 1 [file 12870_2025_6347_MOESM1_ESM.docx]

**SUPPORTING INFORMATION**

**Seed treatment with macroalgal-derived fucoidan and nanohydroxyapatite mitigates *Fusarium falciforme* ASU26 infection in faba bean: Insights from morphological, physiological, anatomical, and FT-IR analyses**

**Mohamed Gomaa*, Eman S. E. Aldaby, Ghada Abd-Elmonsef Mahmoud**

**Botany & Microbiology Department, Faculty of Science, Assiut University, 71516, Assiut, Egypt**

*** Corresponding author**

**Dr. Mohamed Gomaa**

**Botany and Microbiology Department, Faculty of Science, Assiut University, 71516 Assiut, Egypt**

**e-mail: m_gomaa@aun.edu.eg**

**https://orcid.org/0000-0003-1544-3042**

A

E

D

C

B

**10 mm**

F

**Fig. S1:** (A-E) *Liagora viscida* (A), (B) *Liagora viscida* morphology; (C), (D) *Liagora viscida* cortical filaments (E) *Liagora viscida* Carpogonium. (F) *Sirophysalis trinodis*

**Fig. S2:** Phylogenetic tree based on ITS sequences of rDNA of *F.* *falciforme* MK507957 aligned with closely related strains accessed from the GenBank and *Clavispora lusitaniae* (JF433045) represents an outgroup strain.

**Fig. S3:** Transverse sections of 2-weeks old faba bean roots infected with *Fusarium falciforme* ASU26 after different seed treatments. (a) non-infected control, (b) infected control, (c) fucoidan (250 µg mL^−1^), (d) fucoidan (500 µg mL^−1^), (e) nano-hydroxyapatite (250 µg mL^−1^), (f) nano-hydroxyapatite (500 µg mL^−1^), (g) fucoidan + nano-hydroxyapatite (250 µg mL^−1^), (h) fucoidan + nano-hydroxyapatite (500 µg mL^−1^). Scale bars (200 µm).

**Fig. S4:** Normalized FT-IR spectra of 2-weeks old faba bean roots infected with *Fusarium falciforme* ASU26 after different seed treatments. C-: non-infected control, C+ infected control, FUC: fucoidan, nHA: nano-hydroxyapatite. The numbers 1 and 2 indicated concentrations of the compounds as 250 and 500 µg mL^−1^, respectively.
